# Publisher Correction: Spontaneous center formation in Dictyostelium discoideum

**DOI:** 10.1038/s41598-020-61786-6

**Published:** 2020-03-17

**Authors:** Estefania Vidal-Henriquez, Azam Gholami

**Affiliations:** Max Planck Institute for Dynamics and Self-Organization, Am Fassberg 17, D-37077 Göttingen, Germany

Correction to: *Scientific Reports* 10.1038/s41598-019-40373-4, published online 08 March 2019

This Article contains an error in the formatting of Table 1. The correct Table 1 appears below.

**Table 1:** .

*c* = 10 *ҡ* = 18.5 *k*_*t*_ = 0.9 min^−1^ *q* = 4000 *θ* = 0.01	*h* = 5 σ = 0.55 min^−1^ ${\mathcal L}$_1_ = 10 *λ*_*1*_ = 10^−4^ *D* = 0.024 mm^2^/min	*k*_*1*_ = 0.09 min^−1^ *k*_*i*_ = 1.7 min^−1^ ${\mathcal L}$_2_ = 0.005 *λ*_*2*_ = 0.2575 *r* = 10 *µ*m
*a*_*0*_ = 0.04423	*a*_*1*_ = 0.1526	*a*_*2*_ = 328.2
*v*_*c*_ = 0.02 mm/min	*ρ*_*c*_ = 0.6	Δ*γ*_*c*_ = 25.98 nM/mm

