# Supplementary material for: Spontaneous center formation in Dictyostelium discoideum
Source: Sci Rep. 2019 Mar 8;9:3935. doi: 10.1038/s41598-019-40373-4 (PMC6408547; doi:10.1038/s41598-019-40373-4)
Supplement: Supplementary file 10 — SuppInformation [file 41598_2019_40373_MOESM10_ESM.pdf]

Spontaneous center formation in Dictyostelium discoideum

Estefania Vidal-Henriquez[1,*], Azam Gholami [1,+]

[1] Max Planck Institute for Dynamics and Self-Organization, Am Fassberg 17, D-37077 Göttingen, Germany

[*]estefania.vidal@ds.mpg.de
[+]azam.gholami@ds.mpg.de

Supplementary Video S1: Small oscillating cluster. Small group of consecutive cells oscillating synchronously. cAMP concentration in black dots, areas where a cell is located are marked in cyan. $L = 0.15$ mm, $k_e = 5.0$ min^{-1}.

Supplementary Video S2: Large oscillating cluster. Large group of consecutive cells oscillating, a wave developing towards the edges can be observed. cAMP concentration in black dots, areas where a cell is located are marked in cyan. $L = 0.375$ mm, $k_e = 5.0$ min^{-1}.

Supplementary Video S3: System oscillating synchronously. At low degradation rates the system oscillated mostly synchronously and with some phase waves. cAMP concentration in black dots, areas where a cell is located are marked in cyan. Density= $5 \cdot 10^5$ cells/cm^2, $k_e = 4.0$ min^{-1}.

Supplementary Video S4: Target patterns in 1-D. Spontaneous target patterns appearing in a 1-D simulation due to cell distribution. cAMP concentration in black dots, areas where a cell is located are marked in cyan. Density= $5 \cdot 10^5$ cells/cm^2, $k_e = 5.5$ min^{-1}.

Supplementary Video S5: Target patterns in 2-D and cell streaming. Spontaneous target patterns appearing in a 2-D simulation due to cell distribution. Left: cAMP concentration, Right: cell location. Cell movement starts after $t = 50$ min. Density= $4 \cdot 10^5$ cells/cm^2, $k_e = 5.7$ min^{-1}.

Supplementary Video S6: Effect of cell superposition. Without degradation bounded to the cell membrane the centers and streams break apart quickly once cell superposition is added. Left: cAMP concentration. Right: cell location, white locations indicate more than one cell in that grid space. Cell

movement starts after $t = 50$ min. Density$= 5 \cdot 10^5$ cells/cm^2, $k_{eU} = 5.7$ min^{-1}, $k_{eB} = 0.0$ min^{-1}.

Supplementary Video S7: Effect of cell superposition with bounded phosphodiesterase. Adding bounded phosphodiesterase recovers normal stream behaviour. Left: cAMP concentration. Right: cell location, white locations indicate more than one cell in that grid space. Cell movement starts after $t = 50$ min. Density$= 4 \cdot 10^5$ cells/cm^2, $k_{eU} = 2.0$ min^{-1}, $k_{eB} = 6.0$ min^{-1}.

Supplementary Video S8: A lower density signaling center. A group of approximately 18 cells fire synchronously to produce a target center. Red square indicates the approximate location of the firing cells. Left: cAMP concentration, Right: cell location. Density$= 1.25 \cdot 10^5$ cells/cm^2, $k_e = 1.56$ min^{-1}.

Supplementary Video S9: Target and spiral patterns in 2-D and cell streaming. Spontaneous target and spiral patterns appearing in a 2-D simulation due to cell distribution. Left: cAMP concentration, Right: cell location. Cell movement starts after $t = 50$ min. Density$= 4 \cdot 10^5$ cells/cm^2, $k_e = 5.7$ min^{-1}. Circles mark spiral locations.
